# Rhabdomyomas and Tuberous sclerosis complex: our experience in 33 cases

**DOI:** 10.1186/1471-2261-14-66

**Published:** 2014-05-09

**Authors:** Pietro Sciacca, Valentina Giacchi, Carmine Mattia, Filippo Greco, Pierluigi Smilari, Pasqua Betta, Giuseppe Distefano

**Affiliations:** 1 Pediatric Cardiology, AOU Policlinico-Vittorio Emanuele, via Santa Sofia, 78 – 95123 Catania, Italy; 2 Department of Pediatrics, AOU Policlinico-Vittorio Emanuele, via Santa Sofia, 78 – 95123 Catania, Italy; 3 NICU, AOU Policlinico-Vittorio Emanuele, via Santa Sofia, 78 – 95123 Catania, Italy

**Keywords:** Cardiac masses, Rhabdomyomas, Tuberous sclerosis complex, Echocardiography

## Abstract

**Background:**

Rhabdomyomas are the most common type of cardiac tumors in children. Anatomically, they can be considered as hamartomas. They are usually randomly diagnosed antenatally or postnatally sometimes presenting in the neonatal period with haemodynamic compromise or severe arrhythmias although most neonatal cases remain asymptomatic. Typically rhabdomyomas are multiple lesions and usually regress spontaneously but are often associated with tuberous sclerosis complex (TSC), an autosomal dominant multisystem disorder caused by mutations in either of the two genes, TSC1 or TSC2. Diagnosis of tuberous sclerosis is usually made on clinical grounds and eventually confirmed by a genetic test by searching for TSC genes mutations.

**Methods:**

We report our experience on 33 cases affected with rhabdomyomas and diagnosed from January 1989 to December 2012, focusing on the cardiac outcome and on association with the signs of tuberous sclerosis complex. We performed echocardiography using initially a Philips Sonos 2500 with a 7,5/5 probe and in the last 4 years a Philips IE33 with a S12-4 probe. We investigated the family history, brain, skin, kidney and retinal lesions, development of seizures, and neuropsychiatric disorders.

**Results:**

At diagnosis we detected 205 masses, mostly localized in interventricular septum, right ventricle and left ventricle. Only in 4 babies (12%) the presence of a mass caused a significant obstruction. A baby, with an enormous septal rhabdomyoma associated to multiple rhabdomyomas in both right and left ventricular walls died just after birth due to severe heart failure. During follow-up we observed a reduction of rhabdomyomas in terms of both number and size in all 32 surviving patients except in one child. Eight patients (24,2%) had an arrhythmia and in 2 of these cases rhabdomyomas led to Wolf-Parkinson-White Syndrome. For all patients the arrhythmia spontaneously totally disappeared or was reduced gradually. With regarding to association with tuberous sclerosis, we diagnosed tuberous sclerosis clinically in 31 babies (93,9%).

**Conclusion:**

Rhabdobyomas are tumors with favorable prognosis because they frequently do not cause symptoms and they often regress in numbers and size. Nevertheless, due to frequent association with tuberous sclerosis complex and the resulting neurological impairment, the prognosis can result unfavorable.

## Background

Rhabdomyomas are the most frequent cardiac tumors in children followed by fibromas (25% to 30%) [1,2] and less commonly by myxoma, lipoma, teratoma, hemangioma, mesothelioma and Purkinje cell tumour. They are often associated to tuberous sclerosis complex (TSC) [3]. This is an autosomal dominant neurocutaneous disorder that can affect every organ of the body, most commonly the brain, kidney, heart, and lungs [4]. We report our experience with regard to 33 patients with multiple cardiac rhabdmiomata masses, focusing on cardiac outcome and on the association with tuberous sclerosis complex.

## Methods

From January 1989 to December 2012 in the Pediatric Cardiology Unit of University of Catania echocardiography was performed in 14238 patients below twelve months of age using initially a Philips Sonos 2500 with a 7,5/5 probe and in the last 4 years a Philips IE33 with a S12-4 probe.

Every patient if of age, or at least one parent or legal guardian if underage, gave their written informed consent before the patient’s inclusion in the study. The study was conducted in accordance with the Helsinki Declaration, and the study protocol was approved by the (local) Ethics Committee of the Medical University of Catania.

Rhabdomyomas were identified in 33 cases (0,23%). We identified all these patients (Table 1) from the database of our Department of Pediatrics. Clinical features of postnatal examinations were documented from pediatric records and echocardiographic images reviewed from the computer database. Data included age at diagnosis, clinical presentation, physical examination findings (cyanosis, heart murmur, arrhythmia, heart failure), electrocardiogram (ECG), 24-hour ECG recording results, initial and last echocardiography findings (number of rhabdomyomas, location, presence of inflow or outflow tract obstruction, myocardial dysfunction), indication for surgery, outcome (partial or total regression, residual tumors), and follow-up period.

**Table 1:** Clinical presentation – first cardiac medical examination

**Case n°**	**Age at diagnosis**	**Clinical signs at first examination**	**ECG and Holter-ECG**	**Echocardiography at diagnosis**	**Cardiac surgery**
**1**	2 months	Arrhythmia	Atrial and ventricular ectopic beats	Multiple rhabdomyomas	No
			WPW Syndrome		
**2**	1 month	Heart murmur	Normal	Multiple rhabdomyomas	No
**3**	2 months	Heart murmur	Widened QRS	Multiple rhabdomyomas	No
**4**	Fetal	No symptoms but RVOT obstruction	Normal	Multiple rhabdomyomas	No
**5**	1 day	Heart murmur	Normal	Multiple rhabdomyomas	No
		LV obstruction			
**6**	1 day	Heart murmur	Normal	Multiple rhabdomyomas	No
**7**	Fetal	No symptoms	RV hypertrophy	Lobulated mass in LV	No
**8**	Fetal	No symptoms	Normal	Multiple rhabdomyomas	No
**9**	1 month	Heart murmur	RV hypertrophy	Lobulated mass in IVS	No
**10**	1 day	Cyanosis	Atrial and ventricular ectopic beats	Multiple rhabdomyomas	No
		LV obstrution			
**11**	2 months	No symptoms	Atrial ectopic beats	Multiple rhabdomyomas	No
**12**	1 months	No symptoms	RV overload signs	Multiple rhabdomyomas	No
**13**	Fetal	Heart failure Cyanosis	Atrial and ventricular ectopic beats	Multiple rhabdomyomas	Yes
**14**	1 day	Heart murmur	Normal	Multiple rhabdomyomas	No
**15**	7 months	Heart murmur	Atrial and ventricular ectopic beats	Multiple rhabdomyomas	No
			WPW Syndrome		
**16**	1 day	No symptoms	Incomplete right bundle branch block	Multiple rhabdomyomas	No
**17**	9 months	Heart murmur	Normal	Multiple rhabdomyomas	No
**18**	1 day	Arhythmia	Atrial and ventricular ectopic beats	Multiple rhabdomyomas	No
**19**	Fetal	No symptoms (hypokinesia)	Normal	Multiple rhabdomyomas	No
**20**	4 months	Heart murmur	Ventricular ectopic beats and RV overload signs	Multiple rhabdomyomas	No
**21**	Fetal	No symptoms	WPW Syndrome	Multiple rhabdomyomas	No
			IVS overload signs		
**22**	3 days	Heart murmur	Normal	Multiple rhabdomyomas	No
**23**	11 months	No symptoms	RV overload signs	Multiple rhabdomyomas	No
**24**	5 months	No symptoms	Atrial and ventricular ectopic beats	Multiple rhabdomyomas	No
**25**	1 day	Heart murmur	Normal	Multiple rhabdomyomas	No
**26**	9 months	Seizures	Normal	Multiple rhabdomyomas	No
**27**	Fetal	No symptoms	Normal	Multiple rhabdomyomas	No
**28**	2 months	No symptoms	Normal	Multiple rhabdomyomas	No
**29**	Fetal	No symptoms	RV overload signs	Multiple rhabdomyomas	No
**30**	Fetal	Heart murmur	Normal	Multiple rhabdomyomas	No
**31**	7 days	Heart murmur	Normal	Multiple rhabdomyomas	No
**32**	21 days	No symptoms	RV hypertrophy	Multiple rhabdomyomas	No
**33**	Fetal	Heart murmur	Ventricular ectopic beats	Multiple rhabdomyomas	No

Thirty one infants with diagnosis of Tuberous Sclerosis Complex were identified among those who were initially diagnosed having cardiac rhabdomyomas.

We assessed cerebral lesions and development of neurological and skin signs, and eventually kidney and eye involvement in all patients affected by tuberous sclerosis (Table 2). Diagnosis of TSC was based on clinical criteria established in 1998 by the US Tuberous Sclerosis Complex Association [5]. Genetic test for TSC is available on 3 patients (9%) and all are positive.

**Table 2:** General clinical presentation – heart, brain, skin, eye and kidney involvement - and family history

**Case n°**	**Cardiac signs**	**Neurological signs**	**MRI**	**Skin lesions**	**Others**	**Family history**
1	WPW Syndrome	West Syndrome	Cortical tubers	Hypomelanotic maculae	Retinal hamartoma Renal angiomyolipoma	Negative
	Multiple rhabdomyomas			Facial angiofibroma		
2	Heart murmur	West syndrome	Cortical tubers	Hypomelanotic maculae	No	Sister with language disorders
	Multiple rhabdomyomas	Psychomotor delay		Facial angiofibroma		
3	Heart murmur	No	Cerebral white matter radial migration lines	Hypomelanotic maculae	Retinal hamartoma	Positive for TSC
	Widened QRS					
	Multiple rhabdomyomas					
4	Multiple rhabdomyomas	West Syndrome	Cortical tubers	Hypomelanotic maculae	Retinal hamartoma	Negative
		Mental retardation				
5	Heart murmur	No	Normal	No	No	Negative
	Cyanosis					
	Multiple rhabdomyomas					
6	Heart murmur	West syndrome	Cortical tubers	Hypomelanotic maculae	Retinal hamartoma	Negative
	Multiple rhabdomyomas	Drug resistant epilepsy	Subependymal nodules	Facial angiofibroma		
		Psychomotor delay	Cerebral white matter radial migration lines	Forehead plaque		
7	Lobulated mass in LV	West Syndrome	Not performed	Hypomelanotic maculae	No	Negative
8	Multiple rhabdomyomas	Partial seizures	Cortical tubers	Hypomelanotic maculae	Retinal hamartoma	Positive
		Lennox-Gastaut syndrome	Subependymal nodules	Facial angiofibroma		
		Mental retardation	Subependymal giant cell astrocytoma			
		Behavior disorders				
9	Lobulated mass in IVS	No	Not performed	Hypomelanotic maculae	No	Negative
10	Cyanosis	No	Not performed	No	No	Positive for TSC
	LV obstruction					
	Multiple rhabdomyomas					
11	Atrial ectopic beats	West syndrome	Cortical tubers	Hypomelanotic maculae	Renal cysts	Negative
			Subependymal nodules			
	Multiple rhabdomyomas		Cerebral white matter radial migration lines			
12	RV overload signs	West Syndrome	Cortical tubers	Hypomelanotic maculae	No	Negative
	Multiple rhabdomyomas			Facial angiofibroma		
13	Heart failure and cyanosis	West syndrome	Subependymal nodules	Hypomelanotic maculae	No	Negative
	Atrial and ventricular ectopic beats	Behavior disorders	Subependymal giant cell astrocytoma Cerebral white matter radial migration lines			
	Multiple rhabdomyomas	Psychomotor delay				
14	Heart murmur	Drug resistant epilepsy	Cortical tubers	Hypomelanotic maculae	No	Negative
	Multiple thabdomyomas	Mental retardation	Subependymal nodule			
15	Heart murmur	West syndrome	Cortical tubers	Hypomelanotic maculae	Retinal hamartoma, Renal angiomyolipoma	Mother with hypomel. macula
	WPW Syndrome	Behavior and language disorders	Subependymal nodule	Facial angiofibroma		
	Multiple rhabdomyomas		Cerebral white matter radial migration lines			
16	Incomplete right bundle branch block	Partial seizures	Cortical tubers	Hypomelanotic maculae	No	Negative
			Subependymal nodule			
	Multiple rhabdomyomas					
17	Heart murmur	Partial seizures	Cortical tubers	Hypomelanotic maculae	No	Positive
	Multiple rhabdomyomas	Lennox-Gastaut syndrome		Facial angiofibroma		
		Mental retardation				
18	Atrial and ventricular ectopic beats	West Syndrome	Cortical tubers	Hypomelanotic maculae	Renal angiomyolipoma	Negative
	Multiple rhabdomyomas			Facial angiofibroma		
				Forehead plaque		
19	Multiple rhabdomyomas	Partial seizures	Cortical tubers	Hypomelanotic maculae	Retinal hamartoma renal angiomyolipoma	Positive
			Subependymal nodules	Facial angiofibroma		
			Cerebral white matter radial migration lines			
20	Heart murmur	West syndrome	Cortical tubers	Hypomelanotic maculae	Renal cysts	Positive
	Ventricular ectopic beats	Psychomotor delay	Subependymal nodule			
	RV overload signs		Subependymal giant cell astrocytoma			
	Multiple rhabdomyomas					
21	WPW Syndrome	Partial seizures	Cortical tubers	Hypomelanotic maculae	no	Negative
	Multiple rhabdomyomas	Behavior disorders	Subependymal nodule			
22	Heart murmur	No	Cerebral white matter radial migration lines	Hypomelanotic maculae	no	Negative
	Multiple rhabdomyomas					
23	RV overload signs	Drug resistant epilepsy	Cerebral white matter radial migration lines	Hypomelanotic maculae	Retinal hamartoma	Negative
	Multiple rhabdomyomas	Mental retardation		Facial angiofibroma		
				Forehead plaque		
24	Atrial and ventricular ectopic beats	West syndrome	Cortical tubers	Hypomelanotic maculae	No	Negative
	Multiple rhabdomyomas	Psychomotor delay	Subependymal nodules			
25	Heart murmur	No	Cortical tubers	Hypomelanotic maculae	no	Negative
	Multiple rhabdomyomas					
26	Multiple rhabdomyomas	West syndrome	Cortical tubers	Hypomelanotic maculae Forehead plaque	Retinal hamartoma	Negative
			Subependymal nodules			
			Subependymal giant cell astrocytoma			
27	Multiple rhabdomyomas	West syndrome	Cortical tubers	Hypomelanotic maculae	Retinal hamartoma	Negative
		Drug resistant epilepsy	Subependymal nodules	Facial angiofibroma		
		Mental retardation		Forehead plaque		
28	Multiple rhabdomyomas	West syndrome	Cortical tubers	Hypomelanotic maculae	Retinal hamartoma	Negative
		Behavior disorders	Subependymal nodules	Facial angiofibroma		
			Cerebral white matter radial migration lines			
29	Multiple rhabdomyomas	No	Cortical tubers	Hypomelanotic maculae	No	Positive
30	Multiple rhabdomyomas	West Syndrome	Cortical tubers	Hypomelanotic maculae	No	Positive for TSC
			Cerebral white matter radial migration lines			
31	Heart murmur	Drug resistant epilepsy	Cortical tubers	Hypomelanotic maculae	Retinal hamartoma	Negative
	Multiple rhabdomyomas	Mental retardation	Subependymal nodules		Renal angiomyolipoma	
32	Multiple rhabdomyomas	West Syndrome	Cortical tubers	Hypomelanotic maculae	No	Negative
		Mental retardation	Subependymal nodules	Facial angiofibroma		
			Cerebral white matter radial migration lines			
33	Heart murmur	Lennox-Gastaut syndrome	Cortical tubers	Hypomelanotic maculae	Retinal hamartoma	Negative
	Multiple rhabdomyomas	Mental retardation	Subependymal nodules	Facial angiofibroma		
			Cerebral white matter radial migration lines	Forehead plaque		

Follow-up consisted of clinical case review as well as records of investigationswas: subsequent to the diagnosis, all babies were subjected to at least 18 months cardiac follow-up with ECG, color Doppler echocardiography and Holter-ECG every six months. Regarding TSC, under the supervision of a pediatric neurologist, we performed skin and ophthalmological examinations, abdomen ultrasound examination, assessment of occurrence of seizures, brain MRI, aimed at highlighting the typical lesions of tuberous sclerosis complex, and evaluation of mental retardation, psychomotor delay or behavioral and language disorders (the follow-up was between six months and ten years).

## Results

At diagnosis we detected 205 masses: 6 (2,9%) in right atrium, 1 (0,5%) in left atrium, 4 (1,9%) close to valves, 16 (7,8%) in the right infundibulum, 75 (36,6%) in interventricular septum, 45 (22%) in right ventricular wall, 58 (28,3%) in left ventricular wall. In 10 cases (30,3%) rhabdomyomas had been detected antenatally with fetal echocardiography and confirmed at birth. For the other patients, the indications to perform the first echocardiographic assessment were arrhythmias, such as, atrial and/or ventricular ectopic beats in 2 cases (6,1%), in 1 case (3%) the appearance of seizures as infantile spasms, in 2 cases (6,1%) occurrence of cyanosis, in 11 (33,3%) cases presence of a heart murmur, whereas 7 cases (21,2%) were found during routine screening. The mean age in postnatally diagnosed patients was 74,6 days. In 31 patients (94%) we detected multiple masses that led us to define these as rhabdomyomas. Only in 2 patients (6,1%) we observed a single cardiac mass, one intramural in the interventricular septum and one protruding in right ventricular cavity respectively. Of these two, TSC was diagnosed only in one.

In 4 babies (12%) the presence of a mass caused a significant obstruction and/or clinical signs of heart failure and/or cyanosis due to right or left ventricular outflow tract obstruction. Among these 4, in a newborn (3%), with a diagnosis of cardiac masses in the fetal period, surgery to remove the mass was carried out because of severe signs of heart failure and cyanotic spells; a newborn (3%), with antenatal diagnosis of an enormous septal rhabdomyoma associated to multiple rabdomyomas in both right and left ventricular wall, died soon after birth, due to a severe heart failure; whereas in the other 2 patients, with left and right ventricular obstruction respectively, obstructing rhabdomyomas decreased in terms of size without need of medication. Moreover, in another patient (3%) echocardiography revealed only slight hypokinesia without need for medical or surgical therapy. The other 28 patients (85%) did not show signs of hemodynamic impairment.

During follow-up we observed a reduction of rhabdomyomas in terms of both number and size in all 32 surviving patients except in one child. We detected 102 masses: 2 (2%) in right atrium, none (0%) in left atrium, 2 (2%) near the valves, 6 (5,8%) in the infundibulum, 40 (39,2%) in interventricular septum, 25 (24,5%) in right ventricular wall, 27 (26,5%) in left ventricular wall. In 8 cases (24,2%) we found atrial and/or ventricular ectopic beats and in 2 of these cases rhabdomyomas led to Wolf-Parkinson-White Syndrome. For all patients, drug treatment was not believed necessary and the arrhythmia spontaneously healed or was gradually reduced.

With regarding to association with tuberous sclerosis (Table 2), we diagnosed tuberous sclerosis clinically in 31 babies (93,9%) and confirmed diagnosis in 3 patients (9%) by genetic tests. Familial history of the disease was positive in 8 cases (24,2%).

MRI of brain was performed in 30 children and revealed the characteristic lesions of tuberous sclerosis in 29 (96,6%): cortical tubers in 24 patients (80%), subependymal nodules in 16 (53,3%), subependymal giant cell astrocytoma in 4 (13,3%) and cerebral white matter radial migration lines in 12 (40%). Often two or more lesions coexisted in the same patients.

Of 31 affected, 25 (80,6%) developed seizures during follow-up and we cannot exclude symptoms in the future for the remaining. In particular, West Syndrome was present in 16 children (54,3%), Lennox-Gastaut in 3 (9,6%), partial seizures in 5 (16,1%) and drug resistant epilepsy in 5 (16,1%). Moreover mental retardation was present in 9 (29%), psychomotor delay in 5 (16,1%), behavior disorders in 5 (16,1%) and language disorders in 1 (3,2%).

With regrads to skin lesions, we noticed hypomelanotic macules in all 31 patients with tuberous sclerosis (100%), facial angiofibroma in 14 (45,1%) and forehead plaque in 6 (19,3%). Concerning the involvement of other organs, we detected retinal hamartoma in 13 (41,9%), renal angiomyolipoma in 5 (16,1%) and renal cysts in 2 (6,4%).

## Discussion

Cardiac tumours are extremely rare in children (0.027 to 0.17%) [6]. More than one-half of pediatric cardiac tumors are diagnosed within the twelve months of life and are diagnosed both in prenatal and postnatal period [7]. The vast majority of primary cardiac tumours in children are benign, whilst approximately 10% are malignant [8].

Rhabdomyomas are the most common cardiac tumours in children (45%) [9,10].

Echocardiography has been estabilished as the primary diagnostic tool for the evaluation of cardiac tumors in children [11]. Rhabdomyomas appear on ultrasound as round, homogeneous, hyperechogenic, intramural or intracavitary masses, sometimes multiple [12], predominantly localized within the ventricles but can be observed in the atria or caval veins and may lead to obstruction of cardiac valves or inflow/outflow tracts. They are typically asymptomatic but may also cause atrial or ventricular arrhythmias, sinus node dysfunction, heart block and pre-excitation [2,9]. Surgical resection is not usually considered unless they cause severe intractable arrhythmias, valvular obstruction, or congestive heart failure [13]. In any case they are often difficult to remove completely, because they are usually located deep in the myocardium [14].

**Figure 1:**
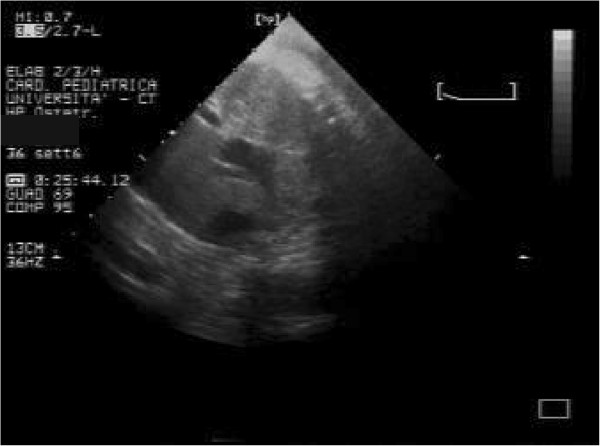
Giant fetal rhabdomyoma.

**Figure 2:**
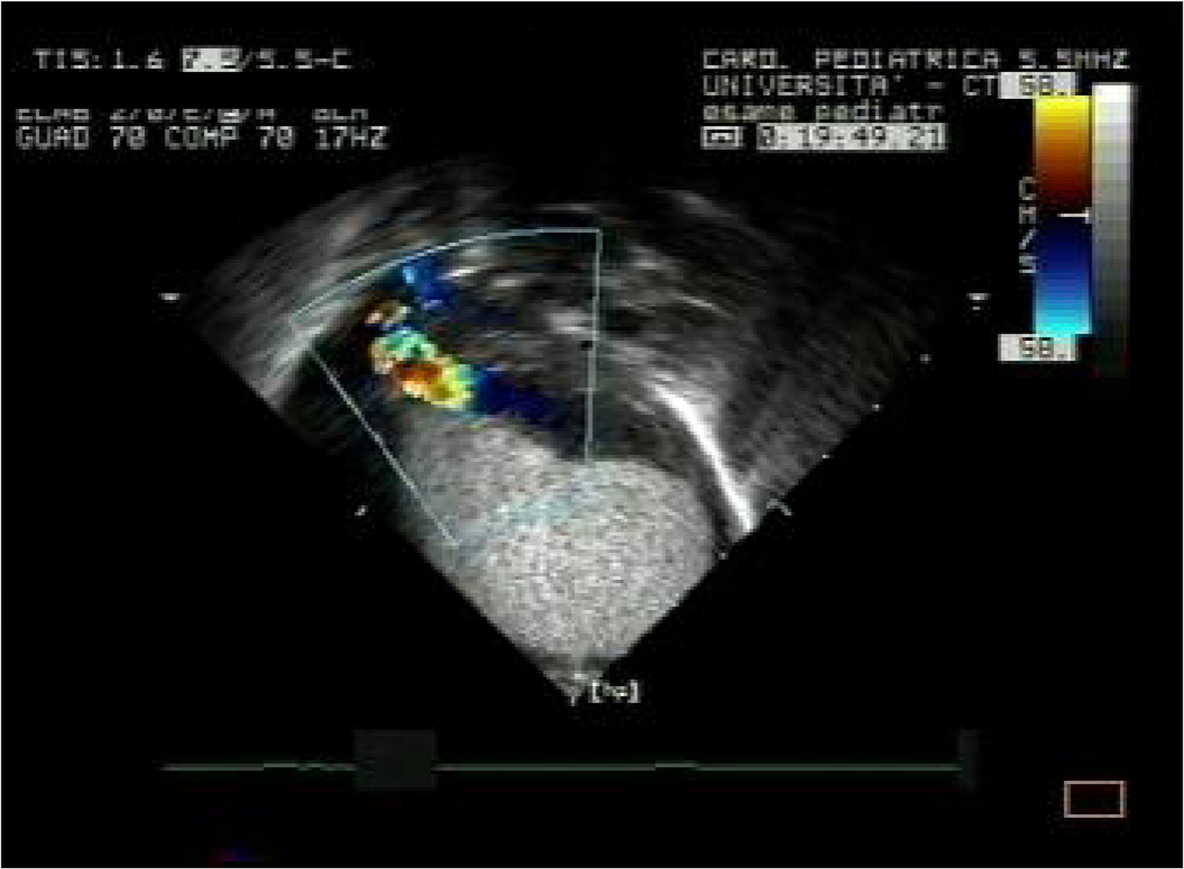
Giant rhabdomyoma in left ventricle.

**Figure 3:**
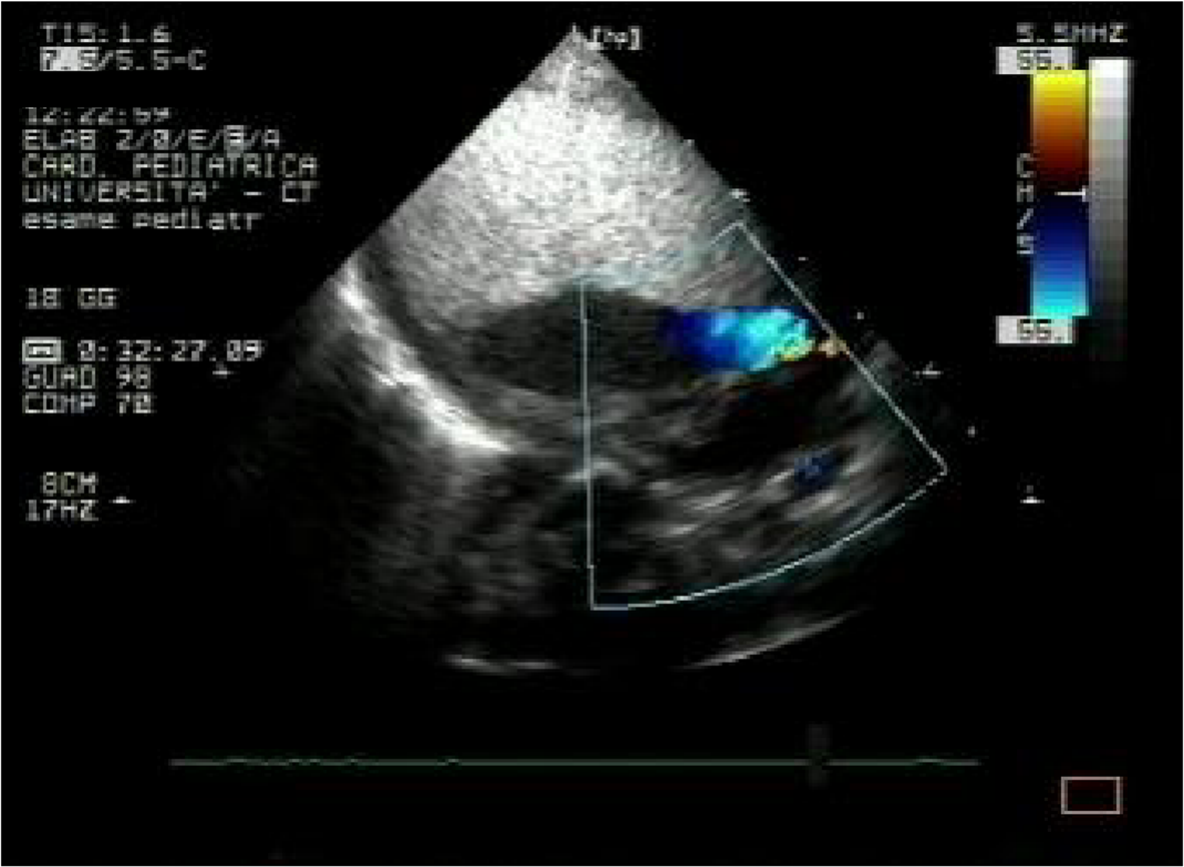
Giant rhabdomyoma – long axis.

In our patients rhabdomyomas were mostly placed in the ventricles (94%), but also in the right atrium (2,9%), left atrium (0,5%) and/or valves (1,9%). Most masses led only to a heart murmur (33,3%) or were identified incidentally (21,2%) during echocardiography, but also were referred rarely due to cyanosis (6,1%) or arrhythmias (24,2%) that totally disappeared or gradually decreased over time. Only one case with signs of heart failure was subjected to surgical resection with good results. Nevertheless, another baby, with severe left ventricular outflow obstruction due to a giant septal rhabdomyomas (Figures 1, 2, 3), died soon after birth.

Congenital cardiac rhabdomyomas represent a condition of particular interest for the researcher due to spontaneous regression of the tumours that occurs in more than one-half of cases [7].

Jozwiak et al. reported that partial resolution of the cardiac rhabdomyomas was reported in 50% of cases and complete regression in 18% and added that these tumors have been reported to grow or to appear de novo in 4% of patients with tuberous sclerosis [15]. Smith et al. showed similar data: regression rates of 60% in preadolescent tuberous sclerosis patients and 18% in adult tuberous sclerosis patients [16,17].

In our experience we noticed involution of rhabdomyomas in all cases with reduction of number and size, with a decrease of all masses in 51,3%, confirming indirectly the histological observations that these lesions regress.

To explain the involution tendency we refer to pathological anatomy: tumours consist of pathognomonic spider cells with centrally placed cytoplasm containing the nucleus and myofibrils radiating to the cell wall [18]. These tumours that seem to originate from embryonic myocytes, represent hamartomas of striated muscular fibers occurring solely in the heart [14].

Immunohistochemical immunoreactivity with ubiquitin, associated with the degradation of myoflaments, progression of cytoplasmic vacuolization, enlargement of glycogen vacuoles, apoptosis and myxoid degeneration are the events providing a plausible explanation for the spontaneous regression of rhabdomyoma [17]. In other words, the involution may be related to the inability of the tumours to divide while the heart chambers grow [19] and this consideration may indicate that some still incompletely identified factors, involved in homeostatic regulation of cardiac biology, could lead to regression of the masses. Infact, after birth, rhabdomyoma cells lose their ability to divide and regression of the tumour in infancy is an expected outcome, regardless of size of the tumour [20-22]. Complete resolution of more than 80% of the tumours may occur during early childhood [23]. Regression may leave a scarred thin chamber wall [7].

The outcome of antenatally detected cardiac rhabdomyomas is also favorable. Once fetal somatic growth is completed, hamartomas lose their mitotic potential and undergo apoptosis [24]. The majority of tumours will regress towards the end of the third trimester although rarely some may continue to grow larger. Despite the expected shrinkage of these tumours, unexpected fetal loss may occur due to arrhythmias or obstruction of blood flow [20-22].

Rhabdomyomas can be sporadic but [2,9] in many cases they are associated with tuberous sclerosis complex (TSC) [25]. This is an inherited multiorgan disease with birth incidence of approximately 1 per 5,000 to 10,000 live births. It is an autosomal dominant neurocutaneous disorder characterized by tumor-like malformations that involve many organ systems, including the brain, heart, kidneys and skin [26].

However, in up to 60% of cases, the disease is related to de novo mutations [27].

It is caused by mutations in either of the two genes, TSC1 or TSC2, which code for the proteins hamartin (chromosome 9q34) and tuberin (chromosome 16p13), respectively, that act as tumour-growth suppressors [28]. Hamartin and tuberin form a complex that activates the GTPase-activating protein Rheb to inhibit the mammalian target of rapamycin (mTOR). mTOR is a highly specific protein kinase that regulates protein synthesis, cellular metabolism, differentiation, growth, and migration. Constitutive activation of mTOR results in the abnormal cellular proliferation and differentiation responsible for the multiple hamartomatous growths throughout the central nervous system, lungs, heart, kidneys, eyes and skin [29].

In our experience rhabdomyomas were associated with tuberous sclerosis in a high number of cases (93,9%) and we found a familial history positive for tuberous sclerosis in about a quarter of cases (25,8%).

Our data mostly conform with the literature in that rhabdomyomas are strong predictors of TSC both when prenatally but especially when postnatally diagnosed [12,30,31].

It is reported that 60-80% of children affected by tuberous sclerosis have cardiac rhabdomyomas, whereas these tumors can be found in only around 20% of adults with tuberous sclerosis [19].

**Figure 4:**
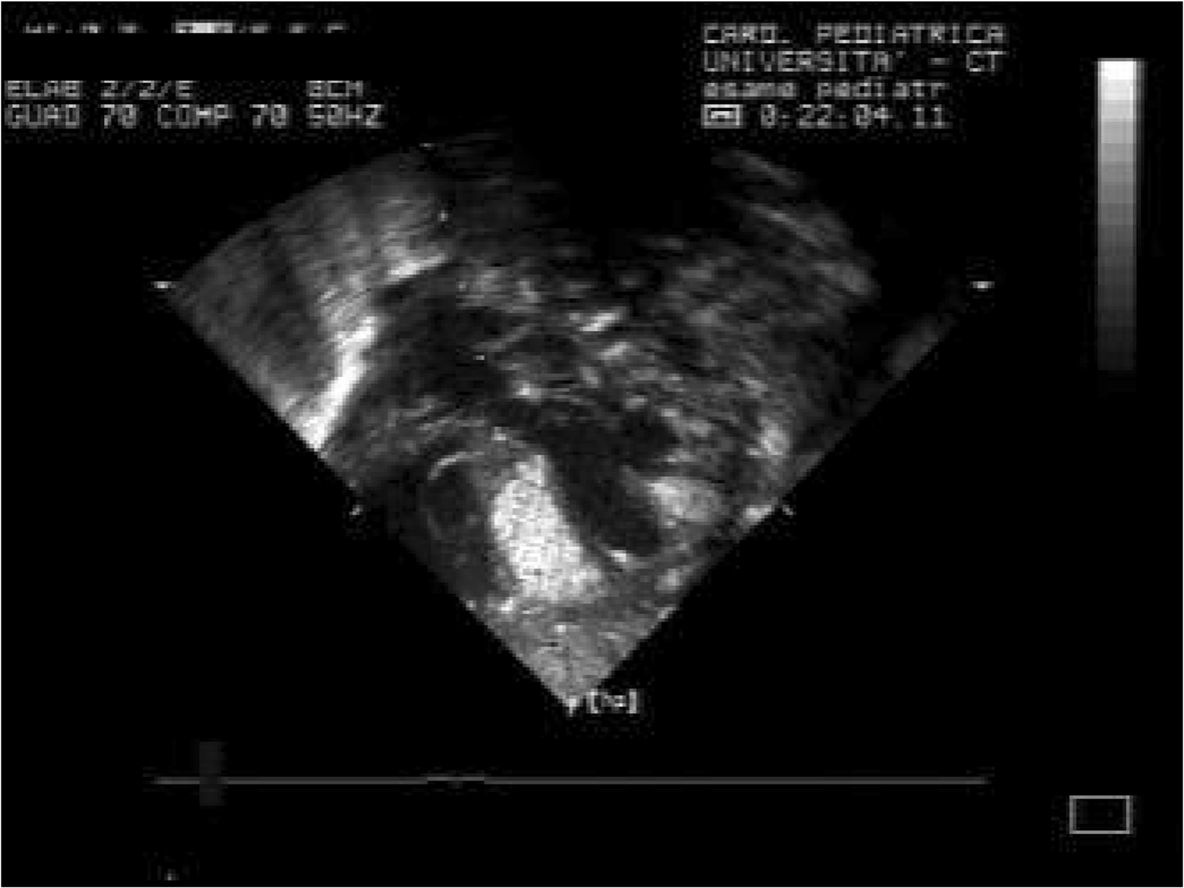
Multiple rhabdomyomas – 4 chambers.

**Figure 5:**
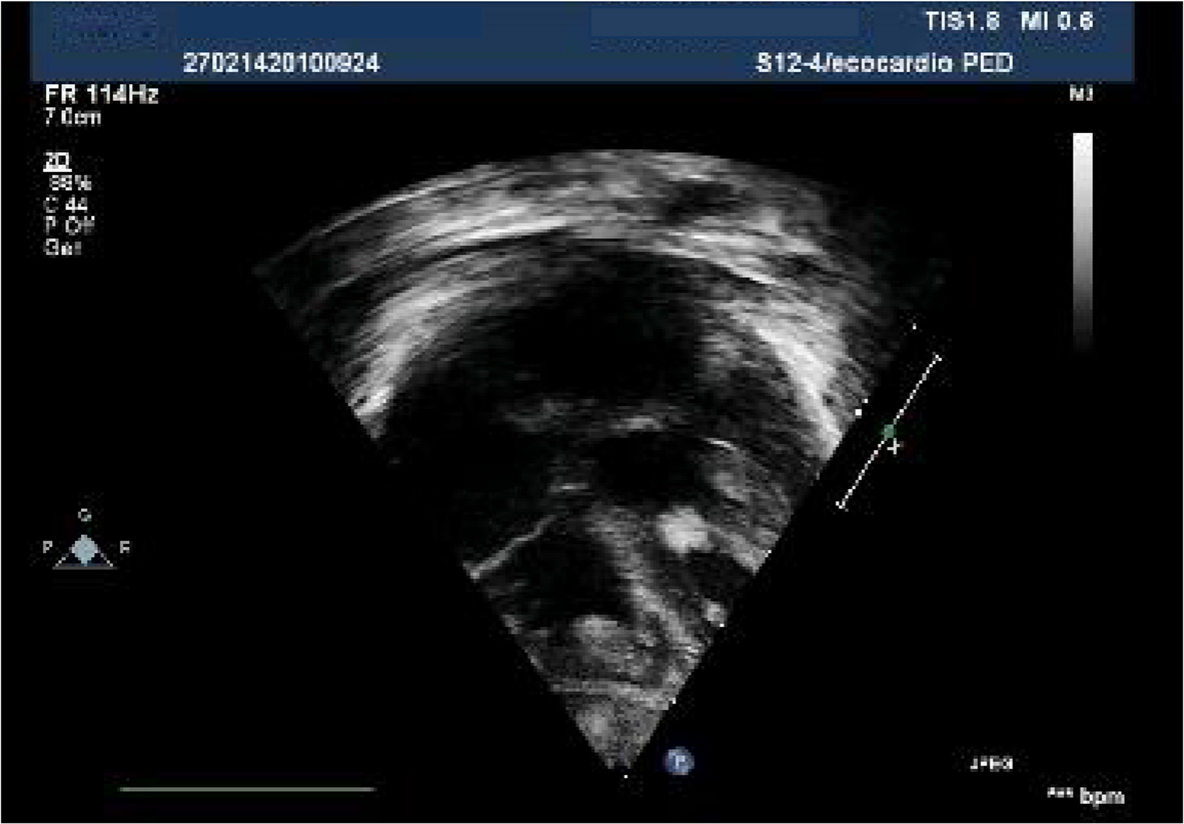
Multiple rhabdomyomas.

In our series almost all children had multiple rhabdomyomas (Figures 4 and 5) with diagnosis of TSC (96,7%). Although the association of multiple cardiac rhabdomyomas with tuberous sclerosis has long been recognized, the association with a single rhabdomyoma is not clear. However, in case of a solitary tumour a careful examination of cardiac chambers should be made in order not to miss smaller lesions elsewhere [32,33].

The diagnosis of TSC is made clinically. A clinical scoring system was developed that divides the diagnostic criteria for TSC into major and minor features. Single or multiple cardiac rhabdomyomas are considered a major feature.

However, the expression and the severity of the disease show substantial variation within, as well as between, families. The classical diagnostic triad of seizures, mental retardation, and facial angiofibromas occurs in fewer than half of the patients [5].

Neurologic manifestations are the most common; 90% of affected people experience seizures, and ∼ 30% to 40% have mental retardation or autism [34,35].

Other signs of tuberous sclerosis include cutaneous lesions, such as, angiofibromas, shagreen patches and hypopigmented macules, brain lesions, such as, cortical/subcortical tubers, subependymal nodules, subependymal giant cell astrocytomas and white matter lesions as well as renal and liver angiomyolipoma, retinal glial hamartomas and cysts in various locations, including the liver, kidneys and pancreas [36].

During follow-up our patients developed seizures in 80,6% of cases and we cannot exclude that the rest of the patients might develop them subsequently. West Syndrome represented the most frequent epilepsy occurring in 54,3% of the cases followed by partial seizures in 16,1%, drug resistant epilepsy (16,1%) and Lennox-Gastaut in 9,6%. Other signs of neurological impairment were mental retardation (29%), psychomotor delay (16,1%), behavior disorders (16,1%) and language disorders in 1 (3,2%). Regarding the brain lesions, they were present in almost all of these patients (96,6%) and they mostly showed cortical tubers (80%). Also the other lesions characteristic of TSC were present: subependymal nodules in few more than half of cases (53,3%), cerebral white matter radial migration lines in less than half of cases (40%) and, rarely, subependymal giant cell astrocytoma in only 13,3% of patients. Often two or more lesions coexisted in the same patient. Finally, about the other lesions of TSC, we found hypomelanotic macules in all, facial angiofibroma in 45,1% and shagreen patches in only 19,3%. We detected retinal hamartoma in almost half of cases (41,9%), renal angiomyolipoma in 16,1% and renal cysts in only 6,4%. We cannot exclude that other lesions may develop over time.

Our data suggest that if the prognosis of rhabdomyomas in usually favorable, it is also true that in cases associated with tuberous sclerosis the general prognosis might be worsened by onset of seizures or lesions in other organs later in life.

## Conclusions

Although there are no consistent guidelines, cardiac monitoring must be carried out for all tuberous sclerosis patients with rhabdomyomas, with serial echocardiography every 6 months and annual Holter monitoring, even if most patients are usually free from cardiac symptoms. Despite the potentially favourable prognosis of patients with cardiac rhabdomyomas, their presence should be sought (by echocardiography) in patients with tuberous sclerosis.

Regarding relation between rhabdomyomas and tuberous sclerosis we must consider that despite the potentially favourable cardiac evolution of patients with cardiac rhabdomyomas, their presence suggests a tuberous sclerosis with a neurological prognosis that is not related to the number or the dimensions of rhabdomyomas.

## Abbreviations

TSC: Tuberous sclerosis complex.

## Competing interests

The authors declare that they have no competing interests.

## Authors’ contributions

PS is the principal author and editor of this article. He has designed, written, reviewed the article and has given final approval of the version to be published. PS also provided the figures from his own collection. VG cowrited the article designed the tables, collected the data and revised the final manuscript and helped with the organisation of references. FG and PlS were involved in the neurological revising of data, PB CM and GD have been involved in interpretation of data. All authors read and approved the manuscript.

## Pre-publication history

The pre-publication history for this paper can be accessed here:

http://www.biomedcentral.com/1471-2261/14/66/prepub

## Work limitations

About the association with tuberous sclerosis complex, we have not got the molecular analysis and/or to have its results for all patients. MRI is not performed in two patients. However we diagnosed tuberous sclerosis complex according clinical signs of disease.
